# Supplementary material for: Ultrafast visualization of incipient plasticity in dynamically compressed matter
Source: Nat Commun. 2022 Feb 25;13:1055. doi: 10.1038/s41467-022-28684-z (PMC8881594; doi:10.1038/s41467-022-28684-z)
Supplement: Supplementary file 1 — Supplementary Information [file 41467_2022_28684_MOESM1_ESM.pdf]

Supplementary Information

Ultrafast visualization of incipient plasticity in dynamically compressed matter

Mianzhen Mo[1⋆†], Minxue Tang[2†], Zhijiang Chen[1], J. Ryan Peterson[1,3], Xiaozhe Shen[1], John Kevin Baldwin[4], Mungo Frost[1], Mike Kozina[1], Alexander Reid[1], Yongqiang Wang[4,5], Juncheng E[6], Adrien Descamps[1,7], Benjamin K. Ofori-Okai[1], Renkai Li[1], Sheng-Nian Luo[2⋆], Xijie Wang[1⋆] & Siegfried Glenzer[1⋆]

[1]*SLAC National Accelerator Laboratory, Menlo Park, California, 94025, USA.*

[2]*School of Materials Science and Engineering, Southwest Jiaotong University, Chengdu, Sichuan, 610031, P. R. China.*

[3]*Physics Department, Stanford University, Stanford, California, 94305, USA.*

[4]*Center for Integrated Nanotechnologies, Los Alamos National Laboratory, Los Alamos, New Mexico, 87545, USA.*

[5]*Materials Science and Technology Division, Los Alamos National Laboratory, Los Alamos, New Mexico, 87545, USA.*

[6]*European XFEL GmbH, 22869 Schenefeld, Germany.*

[7]*Aeronautics and Astronautics Department, Stanford University, Stanford, California, 94305, USA.*

⋆ *To whom correspondence should be addressed. Email: mmo09@slac.stanford.edu; sluo@swjtu.edu.cn; wangxj@slac.stanford.edu; glenzer@slac.stanford.edu;*

† *These authors contribute equally to this work.*

Supplementary Methods

1. Analysis details of Laue diffraction peaks

In this study, the $\{220\}$-family diffraction peaks of the single-crystal Al were analyzed in order to obtain the longitudinal and transverse elastic strains during laser-induced dynamic compression. As indicated in Supplementary Figure 3a, the six $\{220\}$ diffraction peaks are organized into two groups, i.e., the longitudinal strain peak pairs (LSPPs) and the transverse strain peak pair (TSPP), based on the strain information they carry. The LSPPs include two sets of peaks labelled as $\{220\}_1$ and $\{220\}_2$, whereas the TSPP includes the two peaks labelled as $\{220\}_3$. Scattering vectors $\mathbf{k}_\mathrm{l}$ and $\mathbf{k}_\mathrm{t}$ are defined in Supplementary Figure 3a. k is the amplitude of scattering vector, and subscripts l and t correspond to longitudinal and transverse strains, respectively. OA is the projection of $\mathbf{k}_\mathrm{l}$ onto the $\mathbf{k}_x$ direction, and its length is denoted as L. 2θ and γ denote diffraction angle and azimuthal angle, respectively.

For the analysis of the diffraction peaks, we first subtracted the diffraction patterns, both ambient and dynamic, by the preshot background pattern (with both pump and probe switched off). Second, we removed the scattering background signal, arising from the inelastic and multiple elastic scattering[1], in the region that contains the Laue peaks of interest. As an example, we show the step-by-step processed results for the diffraction pattern of the (110) single-crystal Al. We first projected the pattern image in the $\mathbf{k}_x - \mathbf{k}_y$ plane onto the $k - \gamma$ or "radius" – azimuth angle plane (Supplementary Figure 4a), from which we selected six angular regions (non-Laue peak regions) within a designated radial range as the background signal, as shown in Supplementary Figure 4b. At each radius, we took the local average of the selected background signal around the azimuthal area of each peak as the fitted background signal and converted them back to the $\mathbf{k}_x - \mathbf{k}_y$ plane (Supplementary Figure 4c). These fitted background signals, indicated

by the annuli in Supplementary Figure 4c, were then removed from the original diffraction patterns, as shown by Supplementary Figure 4d, before performing the Laue peak analysis.

After the background subtraction, we located the approximate peak positions of the six peaks by fitting the signal in the designated box around each peak with two Gaussian functions (Supplementary Figure 4d). Given these peak locations, we took the intensity lineouts (15-pixel-wide) through the three sets of centrosymmetry peaks. We fitted these lineouts with two Gaussian functions to determine the peak spacings ($2k_{\mathrm{t}}$ and $2k_{\mathrm{l}}$), as well as to extract peak widths and amplitudes. We performed this fitting routine for both ambient and dynamic patterns. The calculation of the longitudinal elastic strain based on Method II (Section 2) will require the knowledge of L and k_{l}. The intensity lineouts of LSPP for finding L are shown in Supplementary Figure 5a, and the intensity lineouts of the TSPP for finding k_{t} are shown in Supplementary Figure 5b.

Supplementary Figure 6 (a) – (c) show the temporal evolution of the radial peak shifts (Δk_{l} and Δk_{t}) of the LSPPs and the TSPP for the (110)-oriented single-crystal Al irradiated at three laser pump fluences. For the LSPPs data, the average results for the two sets of centrosymmetric peaks are shown. Here the positive peak shifts in the reciprocal space are indicative of the decrease in the d-spacing of corresponding lattice planes in the real space, implying the laser-induced compression of the lattice. Likewise, the negative peak shifts imply the expansion of the lattice, which normally occurs when the compression wave is released.

Supplementary Figure 7 shows the temporal evolution of the normalized peak intensities and widths of the same Laue peaks as those shown in Supplementary Figure 6. At 1.2 J cm^{-2}, the intensity of TSPP starts to decay at $\sim$5 ps and reaches $\sim$70% at approximately 10 ps, and then stays constant within the displayed time window ($\leq$70 ps). The LSPP intensity shows no

clear decay before 30 ps; it starts to decay afterwards and reaches $\sim$80% at $\sim$40 ps, and then stays constant for about 20 ps. At 67 ps, the intensity decay of the LSPP and TSPP peaks are observed to be the same. There is no clear change in the peak width of the Laue peaks under this fluence condition.

At 2.2 J cm^{-2}, the intensity decay of TSPP also starts at 5 ps and reaches a plateau of $\sim$50% at $\sim$30 ps. This plateau ends at 50 ps and the intensity declines again. Note that this ending time of the intensity plateau is consistent with the time when the LSPP peak shift returns to zero (Supplementary Figure 6b), implying that this second intensity decay is likely caused by the significant expansion of the sample during the release stage. The LSPP intensity shows a different temporal behavior: after 5 ps, the intensity starts to rise and reaches the peak at $\sim$20 ps; after then, it starts to decay and merges with the TSPP intensity at $\sim$50 ps. We attribute the early-time enhancement of the LSPP intensity to the re-alignment of the mis-oriented grains caused by the initial compression. There is no clear change in the peak widths till the very late time, i.e. $\sim$40 ps, when the peaks start to experience a slight broadening.

At 4.3 J cm^{-2}, the intensities of both LSPP and TSPP follow similar trends to those observed in the case of 2.2 J cm^{-2} except the noted inflection points are advanced in time due to a stronger stress wave driving the sample. The peak widths are observed to broaden at 27 ps, and then progressively increase to a two-fold width at $\sim$70 ps.

2. Strain calculation from measured Laue peak shifts

This section describes our method to calculate the longitudinal and transverse elastic strains based on the UED measurements of the Laue peak positions as detailed in Section 1. It is important to note that the transmission-geometry diffraction patterns taken in the strobo-

scopic manner contain the instantaneous structural information of the dynamically compressed sample, averaged over the electron pulse duration. The extracted peak center obtained from a single Gaussian fitting to the diffraction peak therefore contains the average strain information of the laser-irradiated sample. It is also important to note that: similar to X-ray diffraction measurements, the electron diffraction measurements are only sensitive to elastic strains because the plastic strains do not alter the spacing of lattice planes[2].

Transverse elastic strain. Our pump-probe geometry directly measures the crystal compression normal to the loading direction via TSPP [$(2\bar{2}0)$ and $(\bar{2}20)$]. Under our experimental condition with the loading along the sample normal direction, i.e. [110], the $\{111\}$ slip planes pertinent to plastic deformation are normal to the diffraction planes of TSPP. The resulted crystal rotation occurs in these diffraction planes and will not affect the peak shift of the TSPP[3]. Therefore, the peak shift of TSPP depends only on the transverse elastic strain, $\varepsilon_{\mathrm{t}}^{\mathrm{e}}$, which can be computed using the following equation:

$$\varepsilon_{\mathrm{t}}^{\mathrm{e}} = \frac{k_{\mathrm{t}}^{\mathrm{d}}}{k_{\mathrm{t}}^{\mathrm{a}}} - 1 = \frac{\Delta k_{\mathrm{t}}}{k_{\mathrm{t}}^{\mathrm{a}}}, \tag{1}$$

where $k_{\mathrm{t}}^{\mathrm{d}}$ and $k_{\mathrm{t}}^{\mathrm{a}}$ are the amplitudes of scattered vectors of the transverse strain diffraction peaks of the dynamic and ambient sample (defined in Supplementary Figure 3a).

Longitudinal elastic strain. We use two independent methods to compute the longitudinal elastic strain $\varepsilon_{\mathrm{l}}^{\mathrm{e}}$ based on the longitudinal and transverse strain diffraction peak positions obtained from the UED measurements. The first method (Method I) uses the forward simulation method to directly compare the peak position changes with the experimental input, and the second one (Method II) is based on the analytical equations derived based on the experimental geometry of our diffraction measurements. The results from these two independent methods are in excellent agreement, as shown in Supplementary Figure 8, hence confirming the accuracy of

the inferred longitudinal elastic strains.

We first present the details of Method I, i.e., using the forward simulation of the Laue transmission diffraction patterns[4] to extract the longitudinal elastic strain. In the simulations, we constructed a (110)-oriented Al single crystal with $\pm 2.5^{\circ}$ misorientation to account for the real sample misorientation. The electron source wavelength and the diffraction geometry in the simulation are the same as the UED experiments. Misorientation above $\pm 2.5^{\circ}$ will not affect the final results of the longitudinal elastic strains, except inducing broadening of the Laue peak widths. This is due to the fact that the strains achieved under our experimental condition are relatively small so that a mis-orientation of $\pm 2.5^{\circ}$ is sufficient to capture the compressed lattice planes, as discussed later in Method II.

Supplementary Figure 3 compares the simulated diffraction pattern with the experimental one obtained at the ambient or uncompressed condition. The overall agreement with the experiment on the shapes of Laue peaks justifies the prescribed misorientation of the simulated crystal. Based on this crystal, we applied strains (both $\varepsilon_{\mathrm{t}}^{\mathrm{e}}$ and $\varepsilon_{\mathrm{l}}^{\mathrm{e}}$) to the system and simulated the diffraction patterns to match with the measured peak positions of the $\{220\}$ family at any given time delay from the UED experiments. Here $\varepsilon_{\mathrm{t}}^{\mathrm{e}}$ is a known parameter based on Equation (1), and the best match will therefore yield the solution to the longitudinal elastic strain.

To do this, we varied $\varepsilon_{\mathrm{l}}^{\mathrm{e}}$ within the range from -0.2 to 0.2 at an interval of 0.001. The primary simulation output is the (2θ, γ) positions of the four $\{220\}$ peaks belong to LSPPs. Since the four peaks are centrosymmetric, we select the one residing in the upper-right quadrant of the pattern (Supplementary Figure 3b), with $\gamma = 30^{\circ}$ at ambient condition, to compare with the UED measurement. For experiments, the 2θ coordinate is obtained based on k_{l}, as indicated in Supplementary Figure 3a, which is the average value for the two sets of LSPPs. The γ

coordinate is obtained based on $k_{\rm l}$ and L of LSPPs (Supplementary Figure 3a). Similar to $k_{\rm l}$, the average of the two L for the two sets of LSPPs is used. The details of finding $k_{\rm l}$ and L from diffraction patterns have been described in Section 1. During this matching process, the $\varepsilon_{\rm l}^{\rm e}$ value that gives the minimum difference in (2θ, γ) between simulation and experiment is defined as the best solution. We performed this matching process for all the measurement instants at each pump fluence, and the results are shown in Supplementary Figure 8.

To validate the $\varepsilon_{\rm l}^{\rm e}$ results obtained based on Method I, we applied the analytical method (Method II) to calculate $\varepsilon_{\rm l}^{\rm e}$ that is derived based on the diffraction geometry. Thanks to the narrow energy spread and the low divergence angle of the relativistic electron bunches, UED is only sensitive to lattice planes that are nearly parallel to the electron propagation axis. The inherent misorientation in our single-crystal samples allows the electron beam to capture both the uncompressed and compressed lattice planes in the longitudinal direction. Note that these two lattice planes are not necessary the same planes and only the one parallel to the electron propagation axis will be captured. This rational can be justified by the observation on the peak shifts for the LSPPs of the compressed samples. This is not otherwise expected for a perfect single crystal because the compressed lattice planes would no longer be parallel to the electron propagation axis, and hence the electrons would not be diffracted by the compressed structure (note that grain size broadening can be ignored for the 200-nm thickness Al).

Based on the above argument, the equations are derived for calculating the longitudinal elastic strain. Supplementary Figure 9b presents the Cartesian coordinate system that describes the geometry of the uncompressed and compressed lattice planes. Here we assume that the compressed plane is the diffraction plane, i.e., parallel to the electron beam, and the uncompressed plane is at an angle α with the electron beam. The loading direction and the sample

surface plane are displayed. The loading axis is at an angle φ with the diffraction (compressed) planes. Here $\varphi = 30°$ is known from our loading direction ([110]) and the probed lattice planes, i.e., the $\{220\}$ lattice planes belonging to LSPPs. Point O is defined as the center of the system. Points A′ to D′ are the intercepts of the uncompressed planes with the sample surface and the loading axis; points A to D are the intercepts of the compressed planes with the sample surface and the loading axis. d_0 and d are the respective interplanar spacings for the uncompressed and compressed lattice planes, which are known from the experimental diffraction patterns.

The objective of the following derivations is to solve for ratio m of OB′ over OB (or OC′ over OC), from which the longitudinal elastic strain can be obtained.

The coordinates for points A–D can be readily obtained using their trigonometric relations with distance d and angle φ. Likewise, the coordinates for A′ (z_1, x_1), B′ (z_2, x_2), C′ (z_3, x_3), and D′ (z_4, x_4) are as follows

$$
\begin{aligned}
z_1 &= \frac{d}{2} \times \tan\varphi \times n, x_1 = \frac{d}{2} \times n; \\
z_2 &= -\frac{d}{2 \times \tan\varphi} \times m, x_2 = \frac{d}{2} \times m; \\
z_3 &= \frac{d}{2 \times \tan\varphi} \times m, x_3 = -\frac{d}{2} \times m; \\
z_4 &= -\frac{d}{2} \times \tan\varphi \times n, x_4 = -\frac{d}{2} \times n.
\end{aligned}
$$

Here n is the ratio of OA’ over OA, which is known from the measured $\varepsilon_{\mathrm{t}}^{\mathrm{e}}$ based on the following relation:

$$
n = \frac{1}{1 - \varepsilon_{\mathrm{t}}^{\mathrm{e}}}. \tag{2}
$$

Given the ratio n, the coordinates for points A′ and D′ can be obtained accordingly. The coordinates for points B′ and C′ are still unknown because ratio m is not available yet.

Based on the coordinates of points A′, C′ and D′, the spacing between the two parallel lines A′B′ and C′D′, namely d_0, is given by:

$$\frac{|s \times z_1 - x_1 - s \times z_4 + x_4|}{\sqrt{s^2+1}} = d_0, \tag{3}$$

where s is the slope of the two parallel lines,

$$s = \frac{x_4 - x_3}{z_4 - z_3} = \frac{n - m}{n \tan\varphi + m/\tan\varphi}. \tag{4}$$

Substituting the coordinates of x_1, z_1, x_4 and z_4 into Equation (3), and moving the common factor d to the right-hand side, we have

$$\frac{n \times (s \times \tan\varphi - 1)}{\sqrt{s^2+1}} = \frac{d_0}{d} = \frac{k_{\mathrm{l}}^{\mathrm{d}}}{k_{\mathrm{l}}^{\mathrm{a}}} = \frac{\Delta k_{\mathrm{l}}}{k_{\mathrm{l}}^{\mathrm{a}}} + 1. \tag{5}$$

Equations (4) and (5) will allow us to solve for ratio m. Once ratio m is known, the longitudinal elastic strain ($\varepsilon_{\mathrm{l}}^{\mathrm{e}}$) can be obtained using the following equation:

$$\varepsilon_{\mathrm{l}}^{\mathrm{e}} = 1 - \frac{1}{m}. \tag{6}$$

The results of $\varepsilon_{\mathrm{l}}^{\mathrm{e}}$ obtained based on Method II are shown in Supplementary Figure 8. They are in excellent agreement with those from the forward simulation method (Method I), therefore confirming the accuracy of the inferred longitudinal elastic strains based on the measured Laue peak positions.

Furthermore, based on Method II, we can estimate the misorientation angle, i.e. the angle between line AB and A′B′, which is required for UED to capture the compressed lattice planes. At the highest strain condition of our experiments, this angle is calculated to be approximately $\pm 2^{\circ}$, which is slightly less than the misorientation of our single-crystal samples. Higher strain will require a higher mis-orientation angle in order to be captured by UED.

Supplementary Notes

3. Effect of stacking faults on diffraction peaks.

Our MD simulations show that there are some fraction of stacking faults formed during the plastic deformation process. In this section, we will evaluate the effect of stacking faults on diffraction peaks.

We first performed x-ray diffraction (XRD) simulations of the compressed sample obtained by MD simulation to check if there will be additional peaks in the diffractograms due to the stacking faults. Supplementary Figure 11a shows the XRD results for the two delays of 0 ps and 42 ps at the highest fluence of 4.3 J cm^{-2}. Note that stacking faults peak near 42 ps and the volume fraction at 42 ps is $\sim$8.9%. No additional peak is observed in the XRD pattern of 42 ps, implying that stacking faults will not lead to additional peaks in our electron diffractograms.

Previous studies show that stacking faults can contribute to peak shift of certain orders of diffraction spots in uniaxial compression[5–7]. For FCC metals containing stacking faults on (111) planes, some (hkl) peaks from a given $\{hkl\}$ family are shifted by the stacking faults (affected components) while others are not shifted (unaffected components). The affected components are characterized by $|L_0| = |h + k + l| = 3J \pm 1$ (J is an integer) whereas the unaffected ones follow $|L_0| = 3J$ (J is an integer)[5,6]. In our case, the $\{220\}$-family peaks follow $|L_0| = 0$, implying that their peak shifts are not affected by the stacking faults.

To confirm this, we performed diffraction simulations of the MD atomic trajectories, from which the effect of stacking faults on the peak shifts can be studied[7]. Electron diffraction simulations were carried out using the code GAPD (GPU-accelerated Atom-based Polychromatic

Diffraction)[8]. Supplementary Figure 11b shows the intensity lineout of the simulated $\{220\}$ spots from the MD atomic trajectory obtained at the delay of 42 ps for 4.3 J cm^{-2}. Following the same XRD analysis by Mishra et al.[7], we generated diffraction intensity results for full structure (orange line), FCC atoms only (green line), and the stacking faults atoms (black line). Despite of the presence of 8.9% stacking faults at 42 ps, there is hardly any peak shift that can be identified by comparing the full structure result with FCC-only result. This confirms that the effect from the stacking faults on the peak shifts of $\{220\}$ peaks in our experiment is negligible.

4. Hydrodynamic modelling of laser ablation.

In this section, we provide additional details on hydrodynamics simulations of laser ablation and discuss the influence of the expansion of ablated surface on the calculated strain results. Supplementary Figure 12 (a) - (c) shows the MULTI-1D simulated mass density as a function of time and space for a 200 nm thick Al irradiated by 20 ps (FWHM), 800 nm laser pulses at three pump fluences of 1.2 J cm^{-2}, 2.2 J cm^{-2} and 4.3 J cm^{-2}, respectively. The simulation results show a clear dependence of the ablation process on the incident pump fluence; the onset time of the front surface expansion starts at $\sim$20 ps for 1.2 J cm^{-2} and is advanced to $\sim$10 ps for 4.3 J cm^{-2}, which leads to the differences in the shock wave propagation and the breakout at the rear surface.

To quantify the effect from the expansion at the front surface, we track the change of mass contained in the region of interest (ROI) with x $\geq$ -20 nm. The selection of 20-nm depth is for the consistency with the piston region thickness in MD simulations. We divided the mass of the ROI obtained at each delay time by the total mass, yielding the time evolution of the mass fraction of ROI, as shown by blue lines in Supplementary Figure 12 (d) to (f). Similar trend is observed for the three fluences: the mass fraction is maintained the same before the onset of

hydrodynamic expansion and then gradually increases with an increment of $\sim$7% by the end of 70 ps. Increasing the incident fluence will advance the onset time for expansion as well as accelerating the increase rate of the mass fraction. The additional 7% of the mass loss due to plasma expansion will not affect significantly the simulated elastic strain results that are shown in Figure 3 of the main text. For instance, MD simulations using a 35-nm front layer ($\sim$17% thickness) as the piston region leads to ϵ_l^e of 0.081 at 35 ps, as comparing to the counterpart of 0.076 for the case with 20 nm thickness. Furthermore, the mass density of the ablated plasma is orders of magnitude lower than the solid density, as shown in Supplementary Figure 12 (a) - (c). This indicates that the ablated mass will form a plasma gas cloud in front of the compressed sample and the electron scattering signal from the plasma gas will lead to an increase of the overall background signal.

5. Effect of laser-induced melting and heating on dynamic compression.

In this section, we will discuss the effect of laser-induced melting and heating on the dynamic compression process. In our experiment, the Al sample is irradiated directly by the laser beam, instead of via an intermediate ablation layer. Before the generation of the compression wave, a certain amount of mass near the front surface of the sample will be molten and removed during the laser ablation process. Given the laser intensities of our experiment ($< 10^{12}$ W cm^{-2}), the mass ablation rate of our sample is expected to be less than 10^5 g cm^{-2} s^{-1}(ref.[9]), which corresponds to a material loss of approximately 20 nm in a time scale of 50 ps. This is consistent with the thickness of the piston region (excluded from strain calculation) that was set up for our MD simulations. There is no clear evidence of significant melting during the full compression process, supported by the absence of liquid ring[10] with the diffraction data (See Figure 2 in the main text). This is consistent with what was found in a recent x-ray free-electron

laser (XFEL) experiment (See ref. 35 in the main text) using the technique of small-angle x-ray scattering (SAXS).

Using a thermal diffusivity of 0.9 $\mathrm{cm}^2\ \mathrm{s}^{-1}$ for Al, the time required for heat conduction through the full thickness of the sample is estimated to be ~270 ps[11], much longer than the transit time (~35 ps) for the longitudinal sound wave traversing the sample. Therefore, the temperature rise of the overall sample before the compression is expected to be small during the time scale of the experiment. Nonetheless, to understand the temperature effect on the elastic strain results, we performed additional MD simulations with two initial sample temperatures of 600 K and 900 K that are below the melting temperature of Al. The preheated samples were applied with the same loading profile as the room temperature (RT) case. The temporal evolution of elastic strains for these two preheated temperatures are shown in Supplementary Figure 13, along with the results of RT condition and UED measurement. The results show that as the temperature increases, the transverse strain increases while the longitudinal strain decreases. However, the overall trends of longitudinal and transverse strains including the onset of plasticity remain similar to those of RT condition. On the other hand, MD simulation results with RT condition show the closet agreement with UED data, implying a minor effect from the thermal heating of the sample in the time scale of experiment.

6. Elastic and plastic strains under uniaxial compression.

The discussion in this paragraph is based on Taylor's theory work on the dislocation dynamics[12]. Under the condition of uniaxial compression, the total longitudinal strain ε_l is responsible for the volumetric change of the sample and is given by $\varepsilon_\mathrm{l} = \varepsilon_\mathrm{l}^\mathrm{e} + \varepsilon_\mathrm{l}^\mathrm{p} = -\Delta V/V_0$, where $\varepsilon_\mathrm{l}^\mathrm{e}$ and $\varepsilon_\mathrm{l}^\mathrm{p}$ are the longitudinal elastic and longitudinal plastic strains, respectively. Superscript e (p) stands for elastic (plastic) strain. In this special case of uniaxial compression, the

total transverse strain equals to zero, namely $\varepsilon_{\mathrm{t}} = \varepsilon_{\mathrm{t}}^{\mathrm{e}} + \varepsilon_{\mathrm{t}}^{\mathrm{p}} = 0$. Therefore, $\varepsilon_{\mathrm{t}}^{\mathrm{p}} = -\varepsilon_{\mathrm{t}}^{\mathrm{e}}$. Since plastic strain can not lead to a net volume change, i.e., $\varepsilon_{\mathrm{l}}^{\mathrm{p}} + 2\varepsilon_{\mathrm{t}}^{\mathrm{p}} = 0$, the longitudinal plastic strain can be determined through $\varepsilon_{\mathrm{l}}^{\mathrm{p}} = 2\varepsilon_{\mathrm{t}}^{\mathrm{e}}$. With this, the sample volumetric change can be recast as the mere sum of elastic strains, namely $\varepsilon_{\mathrm{l}} = \varepsilon_{\mathrm{l}}^{\mathrm{e}} + 2\varepsilon_{\mathrm{t}}^{\mathrm{e}} = -\Delta V/V_0$.

Furthermore, $\varepsilon_{\mathrm{t}}^{\mathrm{e}} = -\varepsilon_{\mathrm{t}}^{\mathrm{p}} = 0$ for purely elastic compression, and $\varepsilon_{\mathrm{l}}^{\mathrm{e}} = \varepsilon_{\mathrm{t}}^{\mathrm{e}}(= -\varepsilon_{\mathrm{t}}^{\mathrm{p}})$ for compression in the hydrostatic limit. It is worth noting that electron diffraction measurements are only sensitive to elastic strains because plastic strains do not alter the spacing of lattice planes[2].

Supplementary Figures

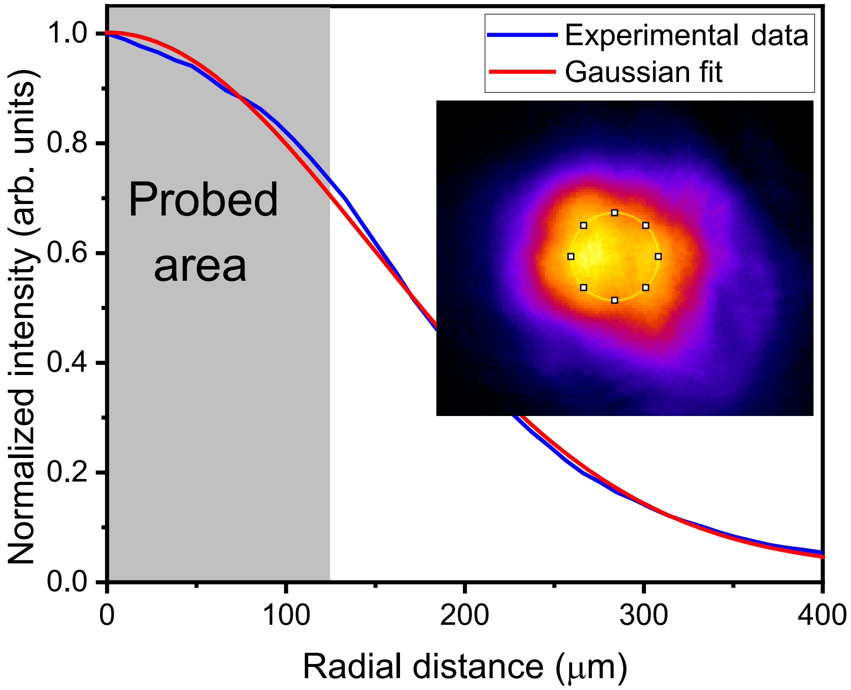

Supplementary Figure 1: Spatial profile of the optical pump pulse for dynamic compression. The image of the focal spot (inset) and its radially averaged intensity lineout (blue solid line). The red solid line is the half Gaussian function fitting to the intensity data. The yellow circle overlaid on the focal spot image indicates the UED probe area that has been projected onto the focal plane of the laser pulse.

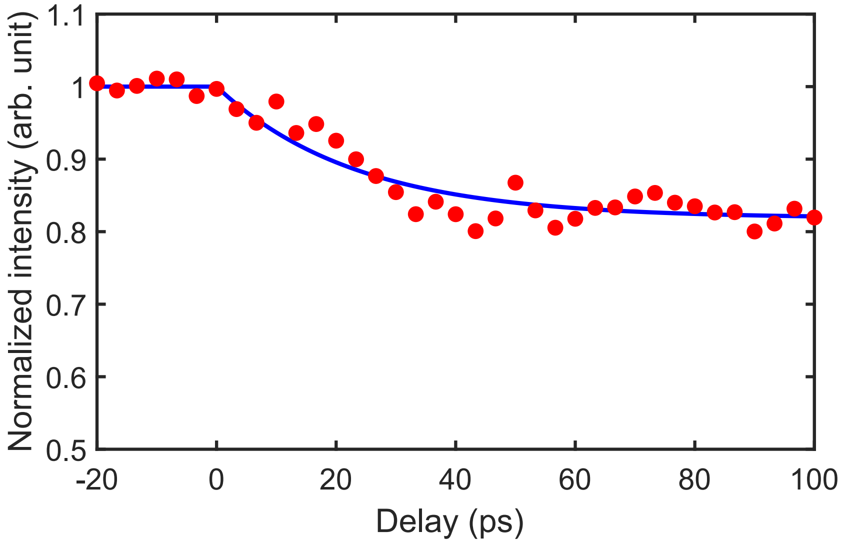

Supplementary Figure 2: Time zero calibration. The red dots are the measured intensity decay of the (533) Laue diffraction peak for the 20-nm-thick polycrystalline gold film (textured) pumped by the 800 nm, 20 ps, laser pulses at a low fluence, as a result of the Debye-Waller factor effect. The blue line is the exponential fitting to the data. Here we define the time zero as the onset time of the decay, which corresponds to the overlap of the leading edges of the optical pump and the electron probe.

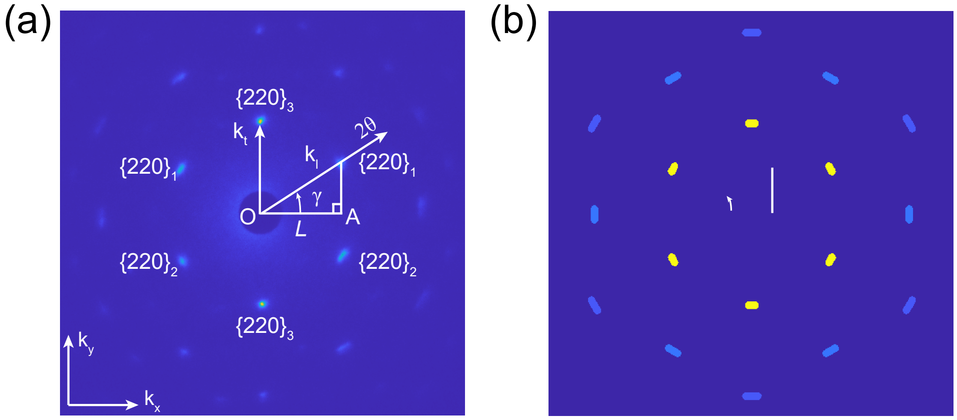

Supplementary Figure 3: Comparison of the measured and simulated diffraction patterns of the (110) single crystal Al at the ambient conditions. (a) UED measurement obtained with an accumulation of 360 shots. (b) Forward simulation with a misorientation of $\pm 2.5°$. The details for the forward simulations of diffraction patterns are described in Section 2. As shown in (a), the six $\{220\}$ peaks are grouped into three pairs, denoted with subscripts 1, 2 and 3. The $\{220\}_1$ and $\{220\}_2$ peak pairs are referred to as the longitudinal strain peak pairs (LSPPs), and the $\{220\}_3$ peak pair, the transverse strain peak pair (TSPP). k is the amplitude of scattering vector, and subscripts l and t correspond to longitudinal and transverse strains, respectively. OA is the projection of k_l onto the k_x direction, and its length is denoted as L. 2θ and γ denote diffraction angle and azimuthal angle, respectively.

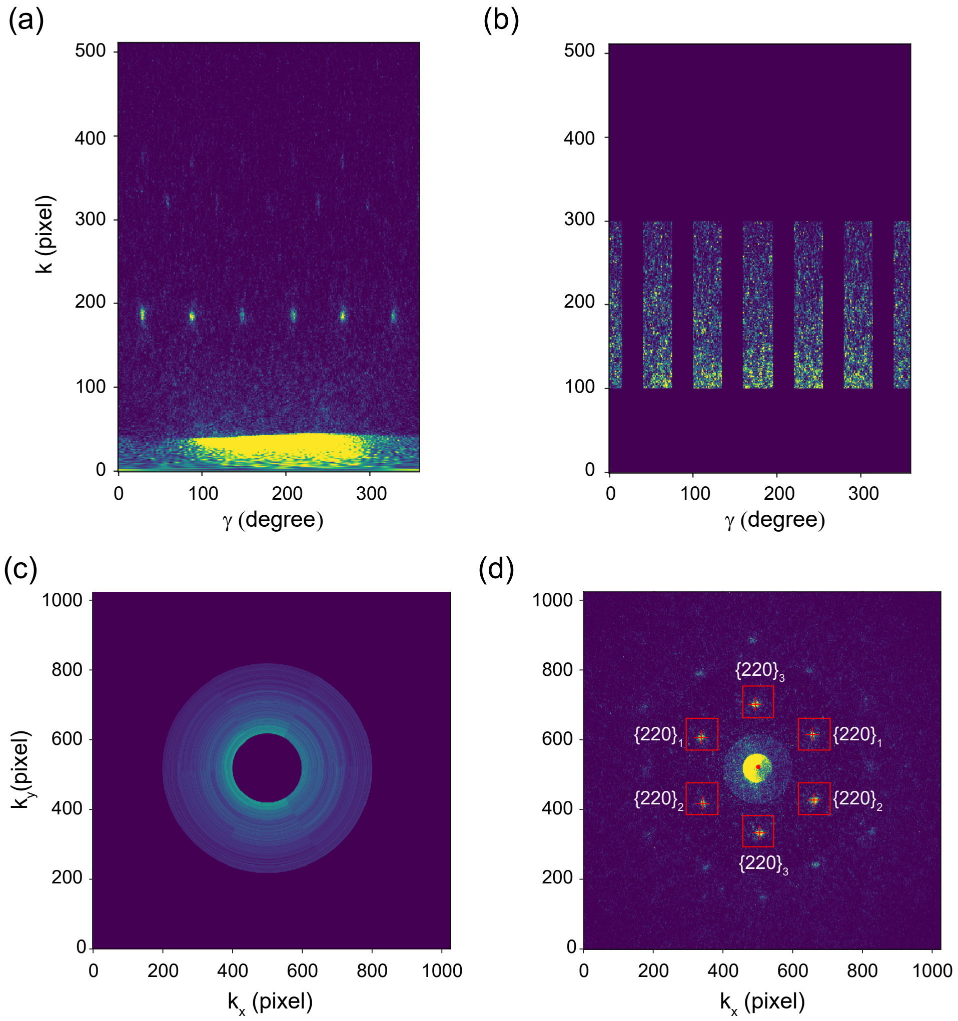

Supplementary Figure 4: Laue peak shift analysis of single-crystal Al under laser-driven compression. (a) – (d) show how the background is removed from the area where the six $\{220\}$ peaks reside on the single-shot diffraction pattern. See the text for more details. In (d), the approximate centroids for the six Laue peaks are marked out by red crosses, based on which the intensity lineouts (15-pixel wide) of the diffraction peaks are obtained and shown in Supplementary Figure 5.

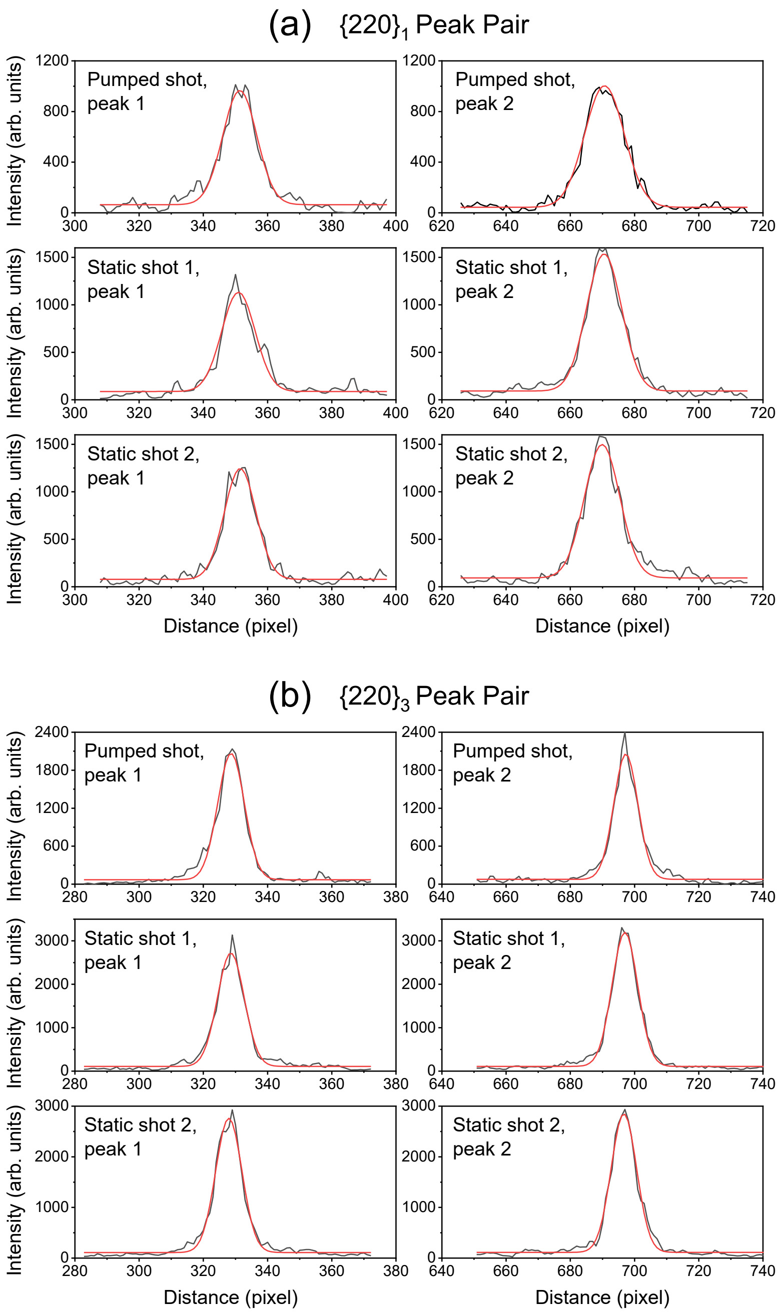

Supplementary Figure 5: Intensity lineouts of Laue diffraction peaks $\{220\}$. (a) The intensity lineouts (black lines) of the $\{220\}_1$ peak pair under shocked and ambient (static) conditions, obtained from the pumped diffraction pattern shown in Supplementary Figure 4D and its reference patterns. (b) Same as (a) but for the $\{220\}_3$ peak pair. In (a) or (b), the top row is for the diffraction patterns from the pumped target, while the bottom two rows are for the two reference (uncompressed or static) patterns of the same target. The intensity lineouts are fit with Gaussian functions (red lines) to extract the peak information such as peak amplitude, position and width.

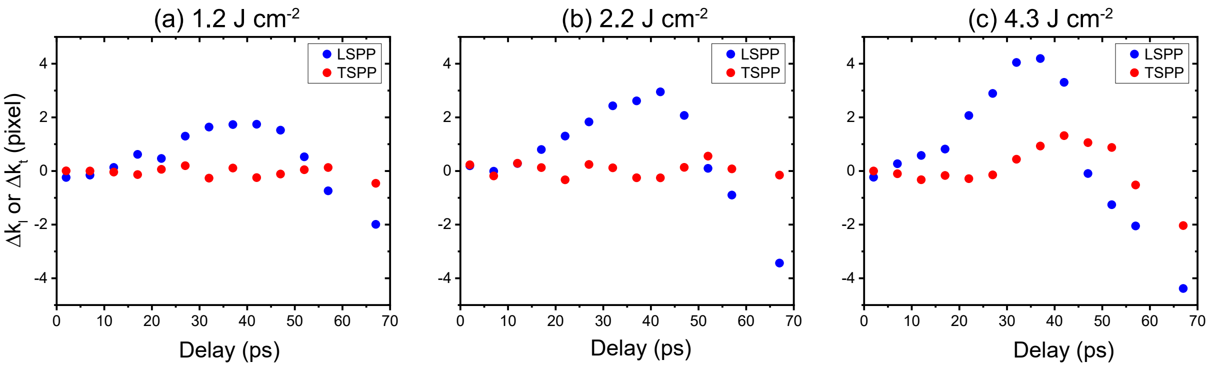

Supplementary Figure 6: Radial Laue peak shifts Δk_{l} (LSPP) and Δk_{t} (TSPP) of the $\{220\}$-family peaks for the (110)-oriented Al under laser-driven compression at incident fluences of (a) 1.2 J cm^{-2}, (b) 2.2 J cm^{-2} and (c) 4.3 J cm^{-2}. Here $\Delta k_{\mathrm{l}} = k_{\mathrm{l}}^{\mathrm{d}} - k_{\mathrm{l}}^{\mathrm{a}}$, and $\Delta k_{\mathrm{t}} = k_{\mathrm{t}}^{\mathrm{d}} - k_{\mathrm{t}}^{\mathrm{a}}$; superscripts d and a refer to dynamic and ambient conditions, respectively. Blue dots are the average results for the two sets of the LSPPs, and red dots, for the TSPP. At each instant, the average value of five pump-probe shots is used.

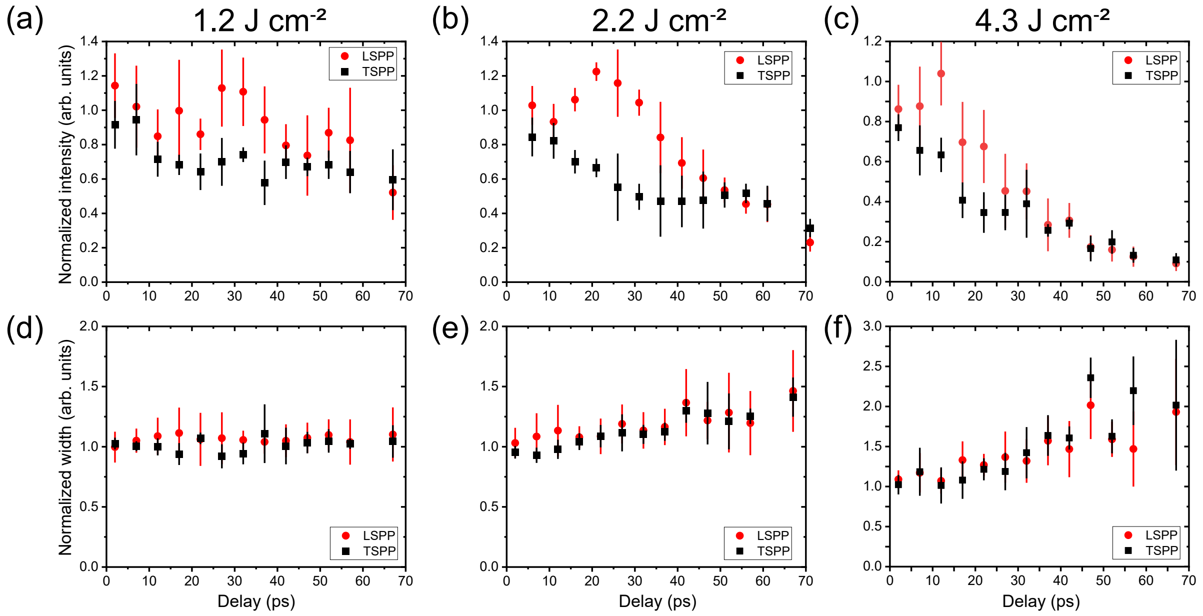

Supplementary Figure 7: Temporal evolution of the normalized intensities (a–c) and widths (d–f) of the $\{220\}$ Laue peak pairs, for incident laser fluences of 1.2 J cm^{-2}, 2.2 J cm^{-2} and 4.3 J cm^{-2}. The peak intensities and widths of each laser shot are normalized to the reference data of the same target at the ambient conditions. Each displayed data point is the average over five pump–probe shots. The error bars represent 1 standard deviation uncertainties. LSPP: longitudinal strain peak pair; TSPP: transverse strain peak pair.

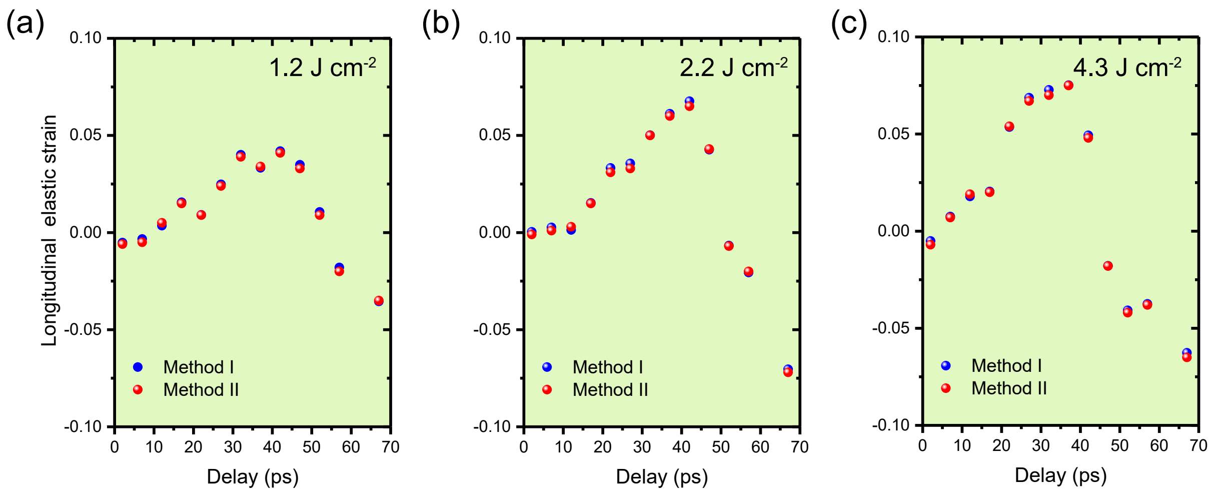

Supplementary Figure 8: Comparison of the longitudinal elastic strains obtained from Method I and Method II for laser-driven compression along [110] at a pump fluence of (a) 1.2 J cm^{-2}, (b) 2.2 J cm^{-2}, and (c) 4.3 J cm^{-2}.

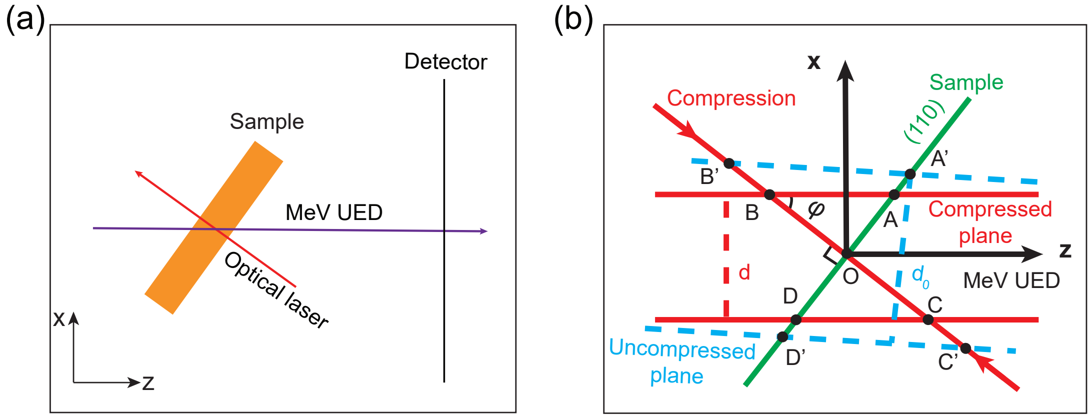

Supplementary Figure 9: (a) Schematic setup for the laser pump–MeV electron probe (diffraction) experiments. (b) Loading-diffraction geometry for an arbitrary lattice plane belonging to LSPPs before and after dynamic compression. Here the dynamic-compressed lattice plane (AB and CD) is parallel to the electron beam direction. After compression, $\mathrm{A'B'C'D'} \rightarrow \mathrm{ABCD}$. Red arrow: compression direction.

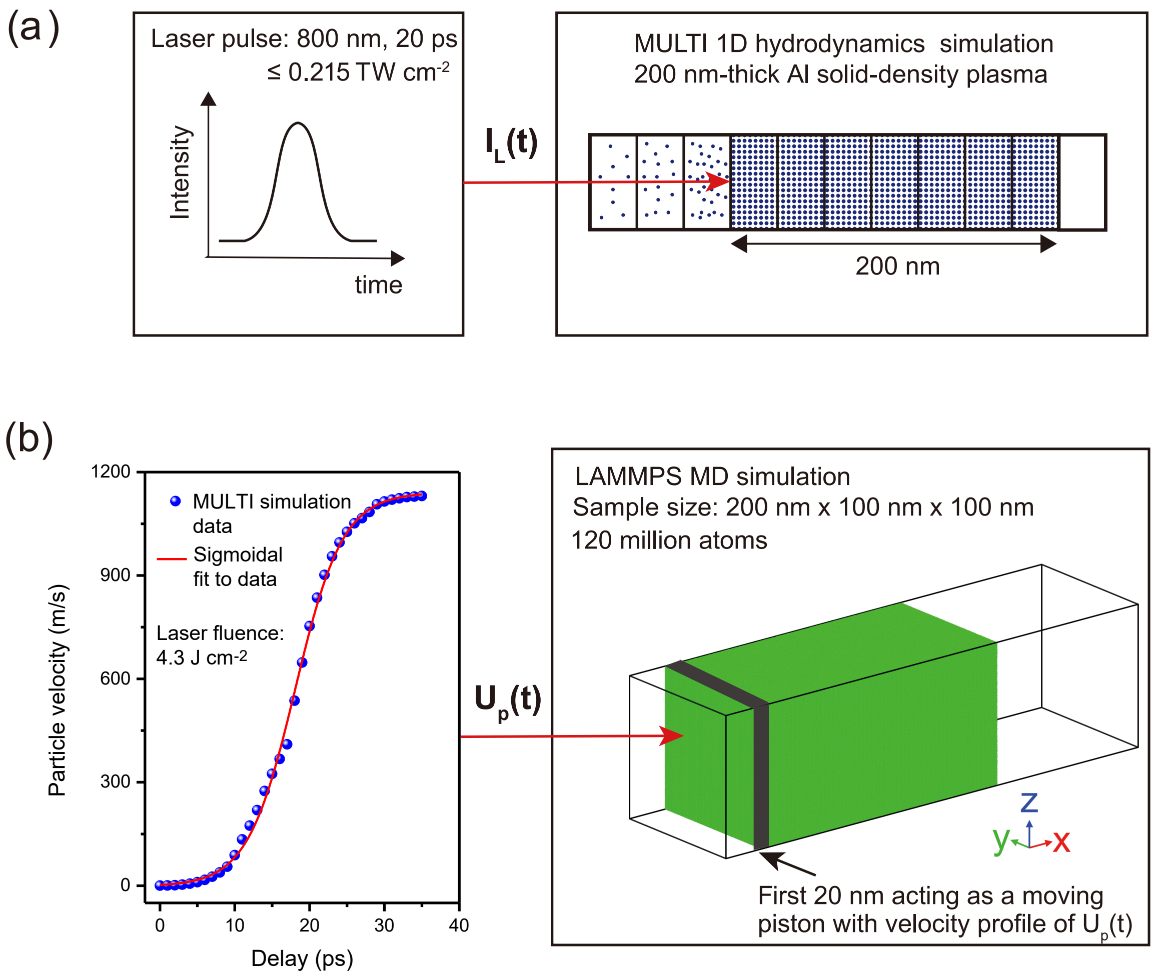

Supplementary Figure 10: The two-step simulation method to simulate laser-driven dynamic compression of aluminum. (a) Step 1: hydrodynamics simulation of the pump laser interaction with the aluminum sample. (b) Step 2: MD simulation of the dynamic compression. The first 20 nm segment on the left of the MD configuration acts as a moving piston to drive the compression wave into the sample. The velocity history of the moving piston is obtained by fitting to the results obtained from the hydrodynamics simulation.

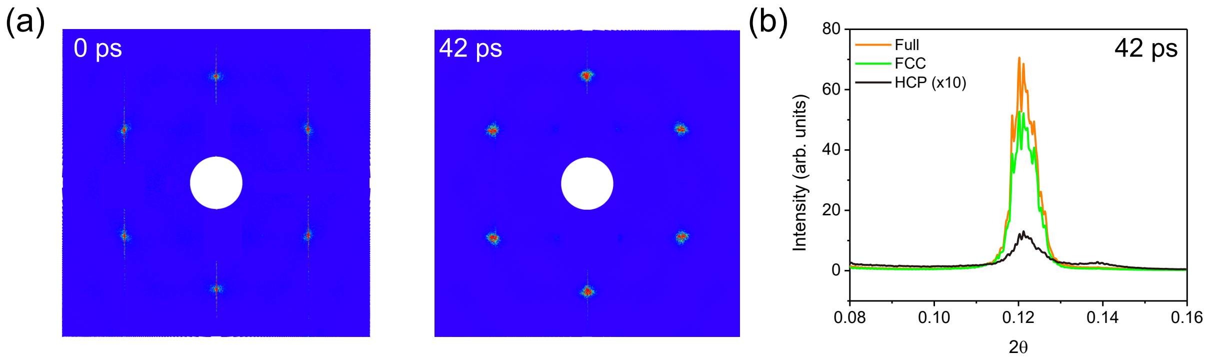

Supplementary Figure 11: (a) XRD simulations of Al-[110] for pump fluence of 4.3 J cm^{-2} at 0 ps and 42 ps. (b) Intensity lineout of the simulated (220) diffraction spot for full microstructure (orange line), FCC only atoms (green line), and HCP atoms of the stacking faults (black line) of the shocked Al at delay time of 42 ps for the pump fluence of 4.3 J cm^{-2}.

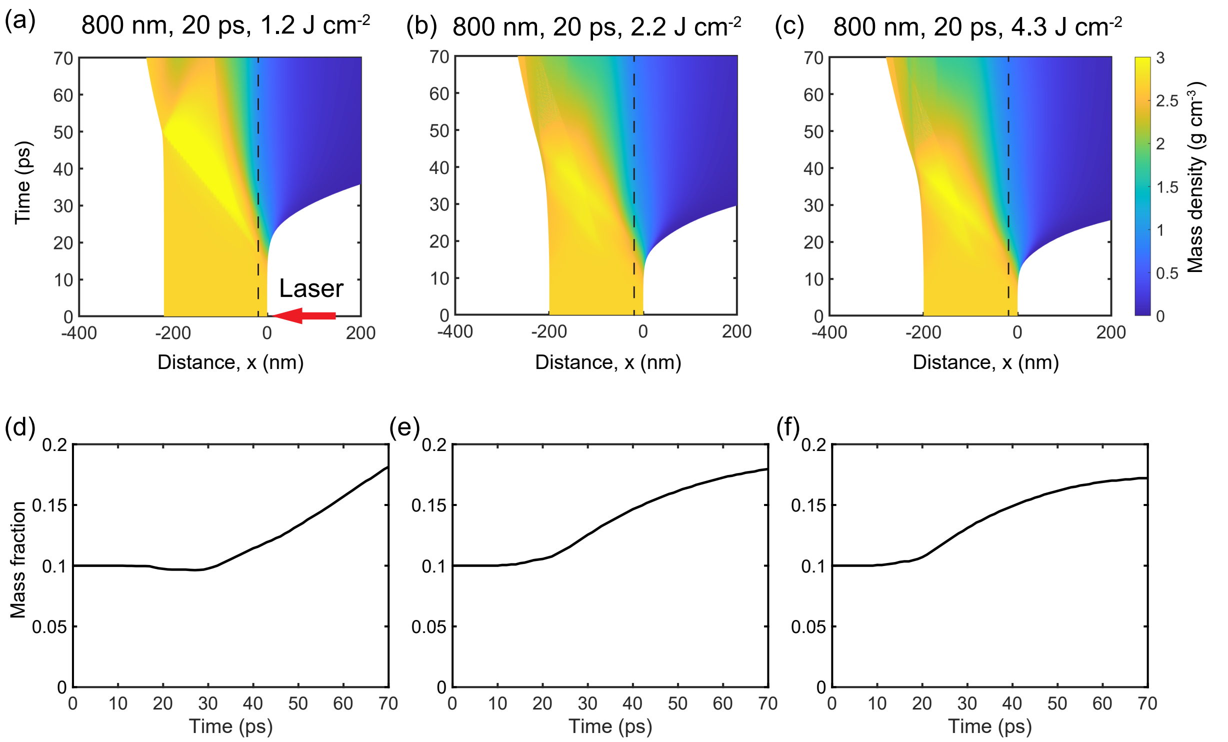

Supplementary Figure 12: MULTI-1D simulation results of the temporal evolution of the mass density distribution for a 200-nm thick Al irradiated by 20-ps (FWHM), 800 nm laser pulses at incident fluences of 1.2 J cm^{-2} (a, d), 2.2 J cm^{-2} (b, e) and 4.3 J cm^{-2} (c, f). The laser impinges on the target at x = 0 nm. The same color axis is applied to all the three false-color images with the representative color bar shown in (c). The vertical dashed lines (x = -20 nm) mark the depth of 20 nm from the target surface. (d) to (f) plot the temporal evolution of the fraction of the mass contained in the ROI with $x \geq -20$ nm for the three respective pump fluences.

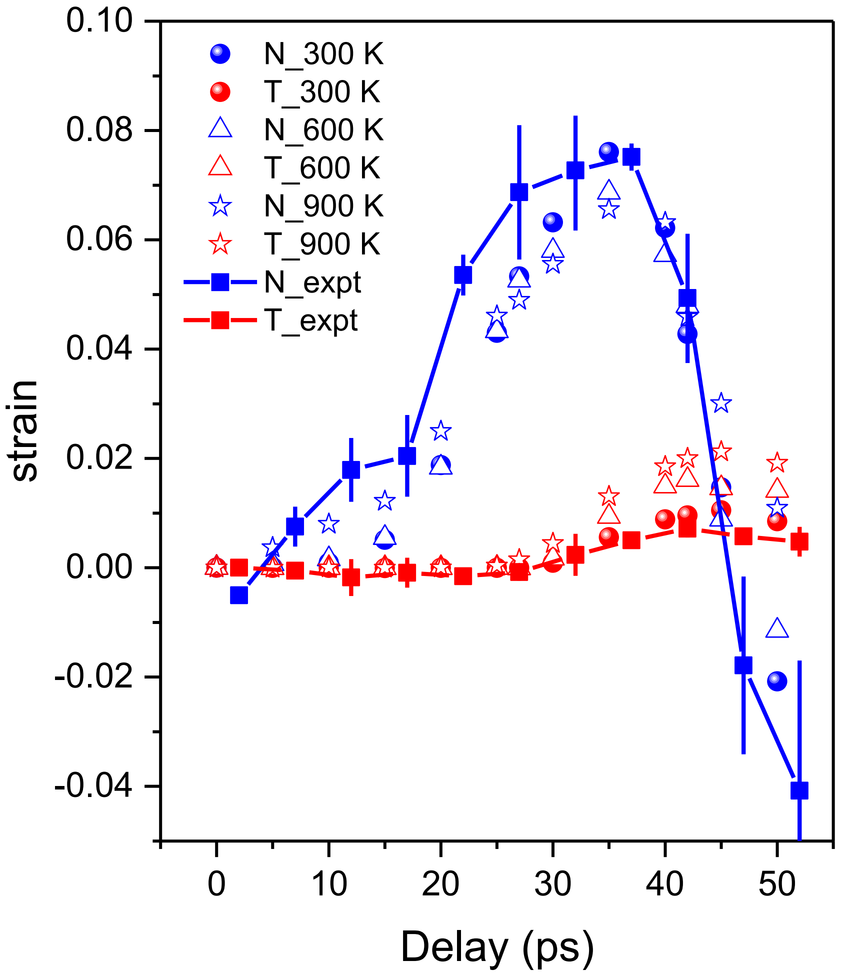

Supplementary Figure 13: MD simulated strain evolution in laser-driven compressed (110) single-crystal Al at different initial bulk temperatures, i.e., 300 K, 600 K and 900 K. The loading condition is the same for the three temperatures and is derived from hydrodynamics simulations with a pump fluence of 4.3 J cm^{-2}. For comparison, UED data (denoted by N(T)_expt) obtained at the same fluence are also presented. The error bars represent 1 standard deviation uncertainties. N denotes normal or longitudinal strain and T denotes transverse strain.

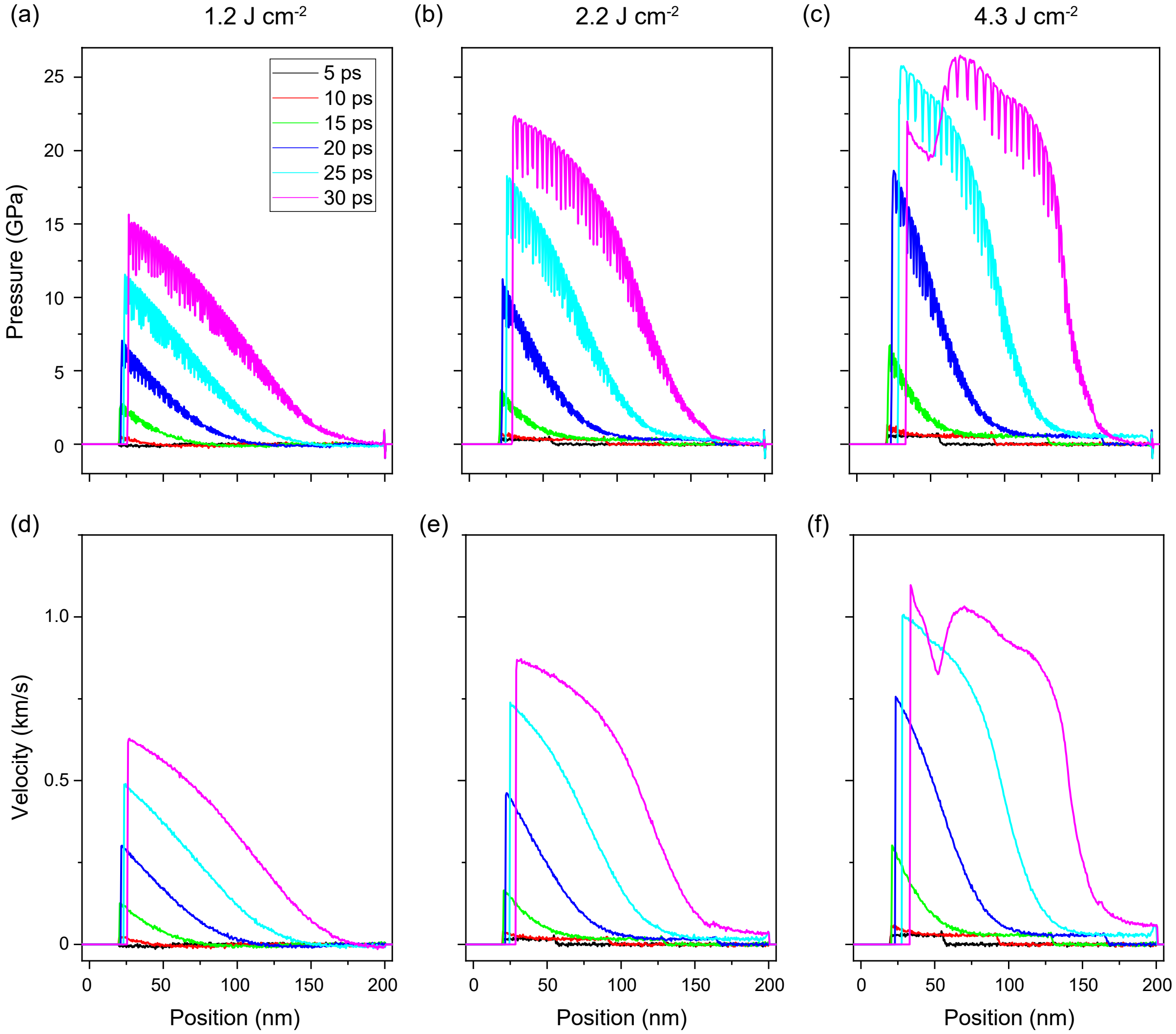

Supplementary Figure 14: MD simulation results of the pressure (a-c) and velocity (d-f) profiles at selective time delays for a 200-nm thick Al irradiated at incident fluences of 1.2 J cm^{-2} (a, d), 2.2 J cm^{-2} (b, e) and 4.3 J cm^{-2} (c, f).

Supplementary References

1. Siwick, B. J., Dwyer, J. R., Jordan, R. E. & Miller, R. Femtosecond electron diffraction studies of strongly driven structural phase transitions. *Chem. Phys.* **299**, 285 (2004).

2. Cullity, B. D. & Stock, S. R. *Elements of X-ray Diffraction* (Addison-Wesley, Prentice Hall, Upper Saddle River, NJ,ed. 3, 2001).

3. Wehrenberg, C. E. *et al.* In situ X-ray diffraction measurement of shock-wave-driven twinning and lattice dynamics. *Nature* **550**, 496 (2017).

4. Gupta, V. K. & Agnew, S. R. Indexation and misorientation analysis of low-quality Laue diffraction patterns. *J. Appl. Cryst.* **42**, 116 (2009).

5. Warren, B. E. *X-ray Diffraction* (Addison-Wesley, Reading, 1990).

6. Sharma, S. M. *et al.* Real-time observation of stacking faults in gold shock compressed to 150 gpa. *Phys. Rev. X* **10**, 011010 (2020).

7. Mishra, A., Kunka, C., Echeverria, M. J., Dingreville, R. & Dongare, A. M. Fingerprinting shock-induced deformations via diffraction. *Sci. Rep.* **11**, 1–12 (2021).

8. E, J. C., Wang, L., Chen, S., Zhang, Y. Y. & Luo, S. N. Gapd: a gpu-accelerated atom-based polychromatic diffraction simulation code. *J. Synchrotron Rad.* **25**, 604–611 (2018).

9. Goldsack, T. J., Kilkenny, J. D. & Rumsbyf, P. T. Determination of mass ablation rates and ablation pressures on spherical targets by ion emission. *J. Phys. D: Appl. Phys* **14**, L47 (1981).

10. Siwick, B. J., Dwyer, J. R., Jordan, R. E. & Miller, R. J. D. An atomic-Level view of melting using femtosecond electron diffraction. *Science* **302**, 1382 (2003).

11. Parker, W. J., Jenkins, R. J., Butler, C. P. & Abbott, G. L. Flash method of determining thermal diffusivity, heat capacity, and thermal conductivity. *J. Appl. Phys.* **32**, 1679 (1961).

12. Taylor, J. W. Dislocation dynamics and dynamic yielding. *J. Appl. Phys.* **36**, 3146 (1965).
